# Middle Miocene long-term continental temperature change in and out of pace with marine climate records

**DOI:** 10.1038/s41598-020-64743-5

**Published:** 2020-05-14

**Authors:** Katharina Methner, Marion Campani, Jens Fiebig, Niklas Löffler, Oliver Kempf, Andreas Mulch

**Affiliations:** 1 Senckenberg Biodiversity and Climate Research Centre (BiK-F), Frankfurt am Main, 60325 Germany; 2 Goethe University, Frankfurt am Main, 60438 Germany; 3 Bundesamt für Landestopografie swisstopo, Wabern, 3084 Switzerland

**Keywords:** Palaeoclimate, Geochemistry

## Abstract

Reconstructing long-term continental temperature change provides the required counterpart to age equivalent marine records and can reveal how terrestrial and marine temperatures were related during times of extreme climate change such as the Miocene Climatic Optimum (MCO) and the following Middle Miocene Climatic Transition (MMCT). Carbonate clumped isotope temperatures (T(Δ_47_)) from 17.5 to 14.0 Ma Central European paleosols (Molasse Basin, Switzerland) display a temperature pattern during the MCO that is similar to coeval marine temperature records. Maximum temperatures in the long-term soil T(Δ_47_) record (at 16.5 and 14.9 Ma) lag maximum ocean bottom water temperatures, lead global ice volume, and mark the initiation of minimum global ice volume phases. The suggested onset of the MMCT, deduced by a marked and rapid decline in Molasse Basin soil temperatures is coeval with cooling reported in high-latitudinal marine records. This is best explained by a change in the seasonal timing of soil carbonate formation that was likely driven by a modification of rainfall seasonality and thus by a major reorganization of mid-latitude atmospheric circulation across Central Europe. In particular, our data suggest a strong climate coupling between the North Atlantic and Central Europe already in the middle Miocene.

## Introduction

The middle Miocene marks an epoch of major global climatic and oceanographic change. The ca. 17 to 15 Ma warm period of the Miocene Climatic Optimum (MCO) interrupted long-term Cenozoic cooling, declining *p*CO_2_ levels and Antarctic ice sheet build-up^1,2^ and contrasts the subsequent middle Miocene Climate Transition (MMCT) that was marked by cooling of high and low latitudes, stabilization of Antarctic ice sheets, major sea level fall and marine biota overturn^3–6^. Long-term paleoclimate records document relatively low Miocene atmospheric CO_2_ concentrations^7,8^, but there is growing evidence for elevated and variable *p*CO_2_ levels of 350 to 630 ppm during the MCO global warm period^9–13^. The MCO may therefore share similarities in the magnitude of global change when compared to the present-day rise in global atmospheric *p*CO_2_, global temperature and decrease in polar ice volume.

In contrast to available marine records, quantitative continental paleoclimate records from the MCO and the subsequent MMCT are sparse, but essential for assessing past global climate change. Paleobotanical studies indicate warmer temperatures during the Langhian (15.97–13.65 Ma) when compared to the Serravallian (13.65–11.60 Ma) on local (e.g. Eastern/Central Paratethys^14^, Denmark^15^) and global scales^16^. Contradictory to the oceanic records, many Central European paleobotanical and mammalian fossil records seem to lack evidence for elevated mean annual temperatures (MAT) during the MCO^17–20^, but indicate decreased temperature seasonality due to elevated cold month temperatures^19^. Indirect evidence for elevated terrestrial temperatures comes from the migration of ectothermic vertebrates to Central Europe during the MCO^21,22^.

Here, we provide a paleosol clumped isotope (Δ_47_) temperature record of the North Alpine Foreland Basin (NAFB) that covers the critical time interval between ~18 and ~14 Ma. Soil development and pedogenic carbonate formation in overbank/floodplain environments in NAFB alluvial mega fans has been extensive, offering valuable insight into the paleoclimate history of central Europe^23–26^. Applying carbonate clumped isotope thermometry offers the unique opportunity to assess the effects of Miocene climate dynamics on mid-latitude continental temperatures in Central Europe and evaluate the interplay of long-term temperature and precipitation patterns during this time of major global climate change.

## Approach and Results

**Figure 1:**
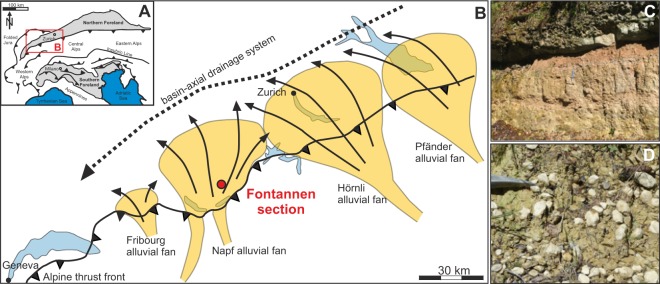
Geological setting. (**A**) Map showing the major alluvial fan systems (yellow) in the Swiss part of the North Alpine Foreland Basin (NAFB) with respect to the present-day thrust front of the Alps during Upper Freshwater Molasse (OSM) deposition. The Fontannen section is located in the proximal part of the Napf alluvial fan system, draining the Central Alps during the OSM phase (modified after ref. ^24^). Maps were generated using CorelDRAW Graphic Suits X5 (https://www.coreldraw.com/en/). (**B**,**C**) Typical paleosols of the NAFB with abundant pedogenic carbonate nodules.

The Fontannen/Napf (Switzerland) section sampled here is part of the Napf alluvial fan, one of the northern Alpine sedimentary mega fan systems consisting of alternating conglomerates and sandstones as well as mudstones with abundant, well-developed paleosols (Fig. 1)^24,27,28^. Individual pedogenic carbonate nodules from mature paleosols (Fig. 1C,D) were sampled along a magnetostratigraphically-dated section^29^, allowing the exact localization of each sample site within the paleomagnetic pattern, and analyzed for clumped isotope thermometry. The original magnetostratigraphy^29^ has been revised^24,30^, assigning the base of the section to chron 5Dr (17.533–17.717 Ma) and the youngest reversal to either chron 5ACr (14.163–14.070 Ma) or chron 5ABr (13.608–13.739 Ma; ages after ref. ^31^). Here, we follow the latter age model (chron 5ABr) as it yields a more conservative approach for the timing and duration of the temperature decline leading into the MMCT. We note that the alternative age model (assignment to chron 5ACr) is equally likely and thus report both models in Table 1 (for detailed analytical descriptions and further information see Material and Method section).

**Table 1:** Soil carbonate T(Δ_47_) and δ^18^O_carbonate_ values, and calculated oxygen isotopic ratios of soil water (δ^18^O_soil water_). T(Δ_47_) errors are calculated as the standard error of the mean.

Sample	Ages	Alternative Ages	T(Δ_47_)	T(Δ_47_) 1σ SE	δ^18^O_carbonate_	δ^18^O_soil water_	δ^18^O_soil water_ 1σ SE
	[Ma]	[Ma]	[°C]	[°C]	[‰, VSMOW]	[‰, VSMOW]	[‰, VSMOW]
MC 946	13.35	*13.97*	16.9	4.1	20.0	−9.9	1.8
MC 956B	14.13	*14.36*	12.7	1.3	19.7	−11.2	0.6
MC 965B	14.48	*14.54*	29.8	2.8	19.7	−7.5	1.1
MC 961B	14.93	14.93	35.1	2.9	20.4	−5.8	1.1
MC 975A	15.36	15.36	28.1	1.7	20.0	−7.6	0.7
MC 981B	15.82	15.82	24.4	1.4	19.2	−9.1	0.6
MC 1011B	16.38	16.38	23.4	2.9	19.5	−9.1	1.2
MC 1003A	16.46	16.46	28.6	4.1	19.9	−7.6	1.6
MC 1001	16.59	16.59	30.6	2.1	19.8	−7.3	0.8
MC 991B	17.38	17.38	25.4	1.6	19.2	−8.9	0.7
MC 987B	17.61	17.61	22.4	3.8	19.1	−9.7	1.6

**Figure 2:**
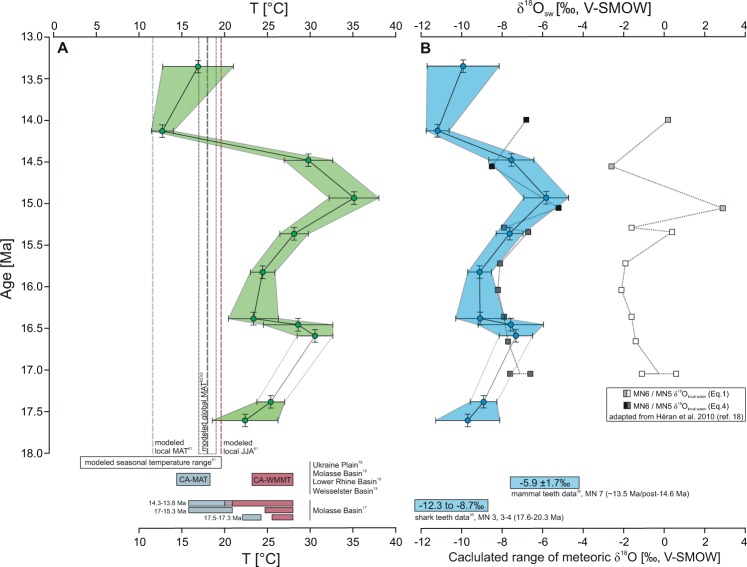
Clumped isotope temperatures and calculated soil water δ^18^O values. (**A**) Temperature reconstructions based on carbonate clumped isotope thermometry (T(Δ_47_)) compared to temperature records from the North Alpine Foreland Basin based on paleofloral data using the coexistence approach^17,19,79^ (blue bars: coexisting approach-mean annual temperature (CA-MAT) and red bars: coexisting approach-warm month mean temperatures (CA-WMMT)) and modeled MATs^61,62,63^. (**B**) Calculated soil water isotopic compositions (δ^18^O_soil water_; blue points) (see SI 1.3 for method description) compared to other meteoric δ^18^O of terrestrial proxy records^18,35,36^ based on δ^18^O values of shark teeth^35^, mammal teeth^36^, and rodent teeth^18^. Note that Héran et al. (2010) (ref. ^18^) used different transfer functions to convert measured δ^18^O values into meteoric δ^18^O values from which we plot the end-member values of their equation 1 (Eq. 1, light grey (MN6) and white (MN5) squares) and 4 (Eq. 4, black (MN6) and dark grey (MN5) squares).

Oxygen and carbon isotope data of the Fontannen section have previously been published^23^ (c.f. Table SI1, Fig. SI1 and in ref. ^23^). Oxygen isotope values (n = 114) of pedogenic carbonate nodules (δ^18^O_carbonate_) are rather constant throughout the section, averaging 19.8 ± 0.4 ‰ (range of 19.0 to 21.4 ‰) with slightly larger variability in the carbon isotope ratios (δ^13^C_carbonate_ = −4.5 ± 1.0 ‰ (−7.0 to −1.9 ‰)). Carbonate clumped isotope (Δ_47_) values range between 0.647 ‰ and 0.703 ‰, translating into T(Δ_47_) temperatures of 35.1 °C to 12.7 °C (Table 1; Fig. 2A,C, Table SI.5). External standard errors (SE) for 4–5 replicate measurements range from ±0.002 ‰ to ±0.011 ‰ (0.9 °C to 4.1 °C). We note that 1 SE values < ~0.0040 ‰ (n = 4) and < ~0.0036 ‰ (n = 5) are below the shot noise limit^32^ of the mass spectrometric set-up (10 acquisitions, consisting of 10 cycles at 20 s integration time each, represent one replicate measurement). Therefore, whenever 1 SE of sample replicates was smaller than 0.0040 ‰, we used the higher shot noise limit of the mass spectrometer as error estimate.

**Figure 3:**
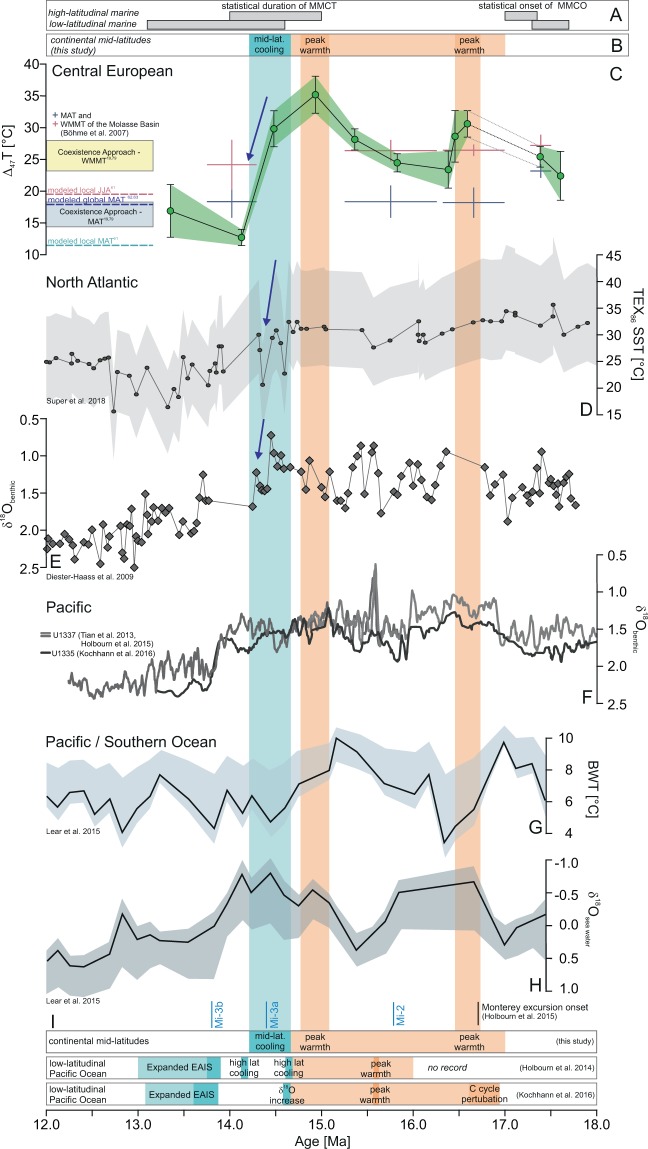
Compilation of terrestrial and marine MCO-MMCT records. (**A**) Statistical onset of the MCO start and the duration of the MMCT^40^, (**B**) Inferred middle Miocene Climatic Optimum (orange) and Middle Miocene Climate Transition (blue) in Central Europe (this study), (**C**) clumped isotope temperature record of the Fontannen section compared to paleofloral-based mean annual temperatures (MAT) and warm month mean temperatures (WMMT)^14,17,19^, and modelled MATs^61–63^ and summer temperatures (JJA)^61^, (**D**,**E**) TEX_86_-based sea surface temperatures^12^ and benthic foraminiferal oxygen isotope values (δ^18^O_benthic_) of ODP site 608 (North Atlantic)^41^, (**F**) low-latidudinal Pacific δ^18^O_benthic_ data^42–44^, smoothed by using the 9pt-average, (**G**,**H**) bottom water temperatures (BWT) and sea water oxygen isotope ratios (δ^18^O_sea water_)^45^, (I) age ranges of temperature reconstructions of the Miocene Climatic Optimum (orange) and the Middle Miocene Climate Transition (blue) by refs. ^6,43^ and this study. Data shown in panels A and D-H is the original work of the authors of the respective papers and thus, the timing of climatic events is independent of the current study (data in panels B,C).

Carbonate T(Δ_47_) at the base of the Fontannen section averages 23.9 ± 4.2 °C (17.61 Ma–17.38 Ma) and increases by ~5.5 °C reaching a first temperature maximum of 29.6 ± 4.6 °C (16.59 Ma–16.46 Ma; Fig. 2A). Subsequently, Δ_47_ temperatures decrease rapidly to 23.9 ± 3.3 °C (16.38 Ma - 15.82 Ma) before they attain a second maximum of 35.1 ± 2.9 °C at 14.93 Ma (mean T(Δ_47_) = 31.0 ± 4.4 °C between 15.4 Ma and 14.5 Ma). Samples younger than 14.5 Ma yield relatively cool temperatures of 12.7 ± 1.3 °C and 16.9 ± 4.1 °C (at 14.13 Ma and 13.35 Ma, respectively), averaging 14.8 ± 4.3 °C. Overall T(Δ_47_) shows warm temperatures (>~24 °C) for samples older than 14.5 Ma with two intervals of elevated temperatures (>~30 °C) at ~16.6 Ma and ~14.9 Ma (“peak warmth”, Figs. 2A and 3B,C). The most remarkable temperature change occurs after the second peak warmth when T(Δ_47_) values drop rapidly from 29.8 ± 2.8 °C (14.48 Ma) to 12.7 ± 1.3 °C (14.13 Ma). Thus, measured soil carbonate formation temperatures more than halved within less than ~350 ka (120 m of section). Applying the alternative age model (assignment to chron 5ACr) this temperature decrease would be even more rapid (less than ~180 ka).

Soil water δ^18^O values (δ^18^O_soil water_), assuming oxygen isotope soil water-carbonate equilibrium were calculated using pairs of T(Δ_47_) and δ^18^O_carbonate_ values and the oxygen isotope equilibrium fractionation equation of ref. ^33^ (updated by the acid fractionation factor of ref. ^34^). δ^18^O_soil water_ values range between −5.8 ± 1.1 ‰ and −11.2 ± 0.6 ‰ (Table 1, Fig. 2B). Since δ^18^O_carbonate_ values do not exhibit large variability across the samples time interval δ^18^O_soil water_ values are mainly controlled by the ambient soil temperature. Pre-14.5 Ma δ^18^O_soil water_ values average −8.1 ± 1.2 ‰ and post-14.5 Ma average −10.6 ± 0.9 ‰, resulting in a decrease in δ^18^O_soil water_ values of −2.5 ± 1.3 ‰. The major temperature drop between 14.5 and 14.4 Ma is accompanied by a δ^18^O_soil water_ value change of −3.6 ± 1.2 ‰.

## Discussion

### Continental soil temperatures and their relation to marine records: MCO

Well-developed paleosol carbonate nodules are abundant throughout the NAFB Fontannen section (Fig. 1C,D; Fig. S1), thus, a lithological change is unlikely to account for the changes in measured soil temperatures. T(Δ_47_) values (12.7 ± 1.3 °C to 35.1 ± 2.9 °C) fall in the range of typical near-surface temperatures indicating that the nodular carbonate formed within the soil column and did not experience any diagenetic alteration and isotopic exchange. Calculated, moderate δ^18^O_soil water_ values concur with other regional δ^18^O_water_ reconstructions^18,35,36^ and permit to exclude strongly evaporitic conditions during carbonate formation due to shallow soil depths (Fig. 2). Absolute T(Δ_47_) are in line with other (semi-)quantitative Central European temperature records^14,17–20,37–39^ (Figs. 2A and 3C) but are now available at a temporal resolution that permits a comparison to marine temperature records. The paleosol T(Δ_47_) record of the Swiss Molasse reveals (1) a characteristic internal temperature structure with two MCO warming periods between 17.4 and 16.6 Ma as well as 15.4 and 14.9 Ma and (2) a very pronounced drop in T(Δ_47_) after 14.5 Ma (Figs. 2A and 3B,C). The two warming peaks with temperatures exceeding 30 °C are about 6 °C warmer than the already elevated MCO temperatures (~24 °C) and delimit the MCO in the Alpine foreland basin record to approximately 16.6 Ma and 14.9 Ma. The onset of the MCO is not clearly resolved due to a lack of data between 17.4 and 16.6 Ma and the exact termination dependent on the preferred age model. Cooling and transition into the MMCT, however, occurred after 14.5 Ma. Notwithstanding, the timing of central European warming agrees with the statistical analysis of a large suite of high-resolution marine oxygen isotope records that bracket the onset of the MCO at high latitudes between 17.4 and 16.9 Ma^40^. The passage into the MMCT is well reflected within the T(Δ_47_) data and cool soil temperatures were attained no later than 14.1 to 13.4 Ma, which concurs well with the reported range of the marine high-latitudinal MMCT^40^ (Fig. 3A–C).

The early stage of the MCO in Central Europe is characterized by a first T(Δ_47_) maximum at around 16.6–16.4 Ma. This warm phase correlates with evidence for a climatic optimum in NW-Europe at 16.5–16.3 Ma^38^ and the beginning of (1) increased benthic and bulk δ^13^C values after 16.7 Ma (Fig. 3I, paralleling the “Monterey excursion”)^41–43^, (2) decreased benthic δ^18^O values (δ^18^O_benthic_) in the North Atlantic^2,41^ (Fig. 3E) and the low-latitudinal Pacific^6,42–44^ (Fig. 3F), as well as (3) increased *p*CO_2_ levels^11,12^ (Fig. SI2). Our terrestrial T(Δ_47_) maximum at ~16.6 Ma lags reconstructed maximum bottom water temperatures (BWT) at ~17.0 Ma by ~400 ka (Fig. 3G), but agrees with minimum sea water oxygen isotope ratios (δ^18^O_sea water_), indicative of minimum global ice volume^45^ (Fig. 3H). Following this first T(Δ_47_) peak, terrestrial temperatures remain low between 16.4 and 15.8 Ma, coinciding approximately with the Mi-2 δ^18^O_benthic_ maximum, a cooling interval with build-up of continental ice sheets^46,47^.

A second warming peak at 14.9 Ma occurs at the end of the MCO immediately before the transition into the MMCT. Warmest soil temperatures in Central Europe (~35 °C) at 14.9 Ma are in temporal agreement with decreased δ^18^O_benthic_ values^41–44^ (Fig. 3E,F), minimum δ^18^O_sea water_ values^45^ (Fig. 3H), and elevated *p*CO_2_^9,12^ (Fig. SI2). Maximum soil T(Δ_47_) (at ~14.9 Ma) lags the BWT maximum (at ~15.2 Ma) by ~300 ka (Fig. 3G) whereas TEX_86_ sea surface temperature (SST) reconstructions^12^ do not display discrete temperature peaks (Fig. 3D). Thus, we observe a consistent (within error of our age model of about ~100 ka) time lag of ~300–400 ka between maximum BWT and mid-latitudinal soil T(Δ_47_), which permits that deep-ocean heat uptake preceding atmospheric warming occurred during the middle Miocene.

### Continental soil temperatures and their relation to marine records: MMCT

After 14.9 Ma, paleosol T(Δ_47_) decreases rapidly with a major temperature decline of ~17 °C occurring within less than ~350 ka (14.48–14.13 Ma; 120 m of section). This mid-latitude Central European cooling recorded in soil carbonates coincides with (1) the statistical onset of the MMCT (Fig. 3A; 14.94 ± 0.15 Ma for high-latitudes and 14.62 ± 0.21 Ma for low-latitudes^40^), (2) decreasing SSTs with earliest minimum SST values^12^ (Fig. 3D), (3) increasing δ^18^O_benthic_ values in the North Atlantic at ~14.45 Ma^41^ (Fig. 3E) and in the low-latitudinal Pacific (Fig. 3F). The latter is earlier and smaller compared to isotope event Mi-3b at ca. 13.9 Ma (global increase in δ^18^O_benthic_ values)^47^ and indicates a first high-latitudinal cooling step at ~14.7 Ma^6,43^ (Fig. 3I). Intital stages of European mid-latitude cooling are hence coeval with marine ice sheet advance in Antarctica (14.7–14.6 Ma)^48^, but precede major ice-sheet expansion in East Antarctica at ca. 13.9 Ma^6,43,45^.

Central European terrestrial T(Δ_47_) and marine proxy records indicate striking similarities over the course of the MCO and MMCT. We observe that the paleosol T(Δ_47_) record and δ^18^O_benthic_ records display the same double-peak structure (described as warming interrupted by global cooling at Mi-2^46,47^) and note that BWT and δ^18^O_sea water_ records (despite larger uncertainties) show similar trends. The Swiss NAFB paleosol record is hence sensitive to temperature change which may have acted as a driver for (polar) ice shield growth and decline and associated changes in δ^18^O_sea water_ and hence δ^18^O_benthic_ values. When compared to ice volume-related changes in oceanic δ^18^O_sea water_ values, our mid-latitude T(Δ_47_) record shows temperature peaks at 16.6 Ma and 14.9 Ma slightly before or at the beginning of δ^18^O_sea water_ minima (i.e. minimum ice volume) at 16.6–15.9 Ma and 14.8–14.0 Ma, respectively^45^ (Fig. 3C,H). It is therefore possible that terrestrial peak warming was immediately followed by periods of major ice loss and minimum ice sheet extent. Consequently, δ^18^O_sea water_ values attain maximum values (corresponding to maximum ice volume) at ~15.4 Ma, following a phase of cooler terrestrial temperatures (16.4–15.8 Ma) and at ~13.8 Ma, following the major soil T(Δ_47_) decline between 14.5 and 14.1 Ma. Collectively, these Δ_47_ data suggest a time lag between terrestrial cooling and (re-)appearance of major ice sheets (on the order of several 100 ka). Despite the diachronous behavior of (marine) localities at different latitudes^40^, as well as dating uncertainties, we observe, however, that (terrestrial) temperatures (1) lag reconstructed BWT by ~300–400 ka, (2) lead maximum global ice volume, and (3) mark the initiation of minimum global ice volume phases.

### Magnitude of post-MCO mid-latitude cooling and paleorainfall patterns

The paleosol temperatures analyzed here show middle Miocene temperature characteristics with overall warming interrupted by Mi-2 cooling and a pronounced decline in T(Δ_47_) at the transition into the MMCT (Fig. 3C). The magnitude of the temperature decrease after 14.5 Ma (~17 °C) is large when compared to the change in temperature known from deep-sea records (1 °C to 6 °C)^1,12,42,45,48^. This amplification of terrestrial temperature change is in itself not surprising; yet the overall magnitude needs particular consideration. The few existing studies of terrestrial MMCT cooling report a minor (1–3 °C)^39,49^, often gradual long-term decrease in temperature^19,50^ or inferred the magnitude of continental temperature decrease by the extinction of temperature-sensitive vertebrates, such as alligators, chameleons and giant turtles (~7 °C at ~14–13 Ma)^21^. Assuming a MAT decrease of ~7 °C during the transition into the MMCT in Central Europe^21^ would require a residual decrease of ca. 10 °C based on the total change in Δ_47_ soil temperatures. We propose that a shift in the seasonality of soil carbonate formation simultaneously with global cooling across the transition into the MMCT best explains the large (~17 °C) and rapid (~350 ka) decrease in Δ_47_ soil temperatures. Carbonate formation seasonality is indirectly related to regional climate change as both precipitation amount and seasonality as well as temperature and evapotranspiration (driven by temperature and wind speed) affect soil drying, which is the main driver in forcing pedogenic carbonate formation^51,52^. As a consequence, recorded Δ_47_ temperatures can be shifted to warmer or cooler periods of the year by changing the prevailing carbonate formation (i.e. dry) season^53–55^.

Other possible mechanisms to increase recorded soil temperatures during the MCO, despite changing ambient temperatures, include a shallowing in soil carbonate formation depth or an increase in temperature seasonality during the MCO warm period with “warmer than average” warm month mean temperatures (WMMT). A shift towards shallower carbonate formation depths^56^ and corresponding less dampened warm (summer) temperatures during the MCO and maximally dampened (approaching MATs) temperatures during the MMCT may (at least partly) account for the large magnitude of the MCO-MMCT temperature change. However, such a shift in soil carbonate formation depth would have to be linked to the prevailing rainfall regime^57^, which in turn argues again for a change in rainfall seasonality and thus in carbonate formation seasonality. The latter has been investigated for the Early Eocene Climatic Optimum, but only a maximum increase of WMMT of ~4 °C has been found for the warmest period in the Cenozoic based on carbonate clumped isotopes and paleofloral analysis^58^. Paleoclimate modelling of the middle Miocene suggest increased temperature seasonality over Europe with slightly warmer summer temperatures over central Europe (~+2 – +4 °C), but reduced winter temperatures (~-2 – −4 °C) compared to control runs^59,60^. Modelled ground temperatures^61^, extracted for the paleogeographic position of the Fontannen section (see Supporting Information SI1.4), show seasonal temperature changes of 16 °C (summer/JJA minus winter/DJF), which is surprisingly similar to our observed temperature changes. However, absolute temperatures are significantly cooler with a MAT of 11.6 °C and a summer (JJA) temperature of 19.6 °C (Fig. SI3), largely underestimating local proxy-based temperatures (T Δ_47_ (this study) and paleoflora data^14,17,19,20,50^). Middle Miocene climate modelling indicates that the deduced soil carbonate temperature swings are in a reasonable range, but further suggests that they might reflect a shift from summer to winter temperatures (full shift of the season), rather than a shift from summer to mean annual formation of carbonate (Fig. SI3).

We cannot fully quantify the individual contributions of temperature seasonality, soil formation depth and changes in carbonate formation seasonality to the detected magnitude of T(Δ_47_) decrease after 14.9 Ma but suggest that carbonate formation seasonality contributed importantly to the recorded T(Δ_47_) decrease during the transition into the MMCT. The large magnitude change towards cooler Δ_47_ soil temperatures suggests that the seasonality of carbonate formation either shifted away from summer months to spring/fall or broadened in duration (now including summer and “non-summer” seasons). As the T(Δ_47_) values presented here are consistent with Central European paleobotanical-based WMMT during the MCO and correspond to mean annual temperatures (MAT) during the MMCT^14,17,19,20,50^ as well as modelled MATs^59–65^ (Figs. 2A and 3C), we propose that MCO Δ_47_ temperatures from the Swiss NAFB reflect summer temperatures exclusively and that a change in rainfall seasonality and thus soil carbonate formation seasonality played an important role in determining T(Δ_47_) values during the MMCT.

The ubiquitous presence of soil carbonates in the Swiss Molasse basin under consistently “wet” MCO conditions (precipitation amounts of ~830–1350 mm/a)^17,19,28,66,67^ argues for pronounced rainfall seasonality in Europe with temporal soil drying and pedogenic carbonate formation. This concurs with paleoclimate modeling studies indicating increased (summer) rainfall amounts during the MCO^59,60,65^. Whereas relative humidity is highest during the summer months in the model runs reflecting MCO conditions^61^, the soil water content is largely reduced, thus supporting soil carbonate formation (Fig. SI3). Overall, during the MCO, carbonate formation in summer months was likely driven by wet-dry cycling under relatively wet conditions with high summer temperatures supporting soil carbonate formation. After the MCO, paleorainfall data indicate a general trend towards decreased rainfall amounts^17,19,28,66,67^ and the development of a summer peak in precipitation across Central Europe^67^. The transition to cool T(Δ_47_) values hence may result from summers that became too wet and too “cold” for further supporting soil carbonate formation exclusively during summer month, meanwhile a general decrease of rainfall amounts promotes carbonate formation in other than summer months. Alternatively, a general aridification (contemporaneous with global cooling after the MCO)^3^ could have broadened the interannual time interval for carbonate formation, being no longer restricted to warm months (as before under wet MCO conditions). Both explanations are in line with decreasing δ^18^O_soil water_ values at the end of the MCO (Table 1), as summer rainfall δ^18^O values are typically higher when compared to non-summer rainfall values^68^.

Changing precipitation amount and seasonality over Central Europe at the onset of the MMCT requires profound reorganization of atmospheric circulation in conjunction with global temperature change. A general poleward shift of the Hadley circulation has been predicted for the MCO^60^ and it has been hypothesized that trade winds (northeasterlies)^22^ or even easterly winds along the Alpine chain^69,70^ were dominating Central Europe during the middle Miocene. If (north-)easterly winds have been prevailing during the middle Miocene^22,69,70^, such air mass presumably brought moist air from the Paratethys mainly during the summer month^69^. With decreasing temperature and/or retreat of the Paratethys, the Central European climate likely became more continental^39,71^ and increased influence of the westerlies may have increased winter precipitation of the Molasse Basin. Alternatively or even in conjunction, more northern trade winds may have been efficiently blocked along the Miocene Alps, forcing major rainout of moisture-bearing air masses during rainy season(s)^72^. As soil temperatures show that Central Europe experienced major cooling and the meridional temperature gradient increased across the MMCT^3^, this trade wind zone may have shrunk to more southern latitudes, now allowing westerlies to be the prevailing winds in Europe. These orogen-parallel winds would not have been as efficiently blocked by the Alps as north-south oriented winds and as a result may have led to reduced precipitation close to the Alpine orogenic front and generally drier conditions (contemporaneous with global aridification). Thus, with the disappearance of the trade winds (northeasterlies), the westerly-dominated wind regime over Central Europe might have been established since ~14.5 Ma, about 3 Ma earlier than previously reported^73^.

Our Swiss Molasse paleosol temperature record shows that mid-latitude continental Europe was not only comparably affected by middle Miocene climate dynamics as the global oceans, but experienced profound changes in temperature and possibly in rainfall patterns (seasonality) within a brief time interval, underlining the sensitivity of continental climates to global changes.

## Conclusions

The NAFB paleosol T(Δ_47_) shows two warming peaks (>30 °C) that are bracketing the MCO with warm season temperatures typically >24 °C. The onset of the MMCT is characterized by a major rapid soil temperature (−17 °C) and δ^18^O_soil water_ (−3.6 ‰) decrease within less than 350 ka (14.48–14.13 Ma) after peak MCO temperatures at ~14.9 Ma. The terrestrial temperature record displays a double-peak temperature structure strikingly similar to marine records, revealing marine isotope events Mi-2 and Mi-3a^47^ in the terrestrial NAFB record. Compared to global ice volume and BWT records^45^, peak soil T(Δ_47_) lags reconstructed maximum BWTs by ~300–400 ka, likely leads maximum global ice volume (by several 100 ka), and marks the initiation of minimum global ice volume phases. Cooler soil temperatures are attained at ~14.1 Ma contemporaneous with Mi-3a and decreased North Atlantic SSTs^12^ and δ^18^O_benthic_ values^41^, indicating a strong coupling between the North Atlantic and Central European climate already in the middle Miocene.

In combination with paleofloral and fossil data, we infer that the NAFB was dominated by warm and wet climates during the MCO, but with pronounced wet-dry cycling to allow soil carbonate formation. These observations are consistent with other global warming intervals such as the Middle Eocene Climatic Optimum or the Paleocene-Eocene Thermal Maximum, showing the intensification of the hydrologic cycle during warm periods^53,74,75^. The large and rapid shifts of Δ_47_ temperatures and soil water δ^18^O values argue for a change in the seasonality of soil carbonate formation that concurred with the onset of global cooling at the MMCT. This implies a modification of rainfall seasonality and thus a major reorganization in atmospheric circulation across Central Europe.

## Method and Materials

### Clumped isotope analyses

Carbonate clumped isotope temperatures (T(Δ_47_)), a proxy for soil carbonate formation temperatures, have been measured at the Goethe University - Senckenberg BiK-F Joint Stable Isotope Facility Frankfurt (Germany) according to analytical outlines provided by ref. ^76^ and ref. ^77^. Each sample was measured with at least 4 replicates and each day 2–3 carbonate reference materials were measured alongside with sample unknowns. Temperatures are calculated using the calibration of ref. ^76^. Replicates over the entire analytical period (2013 to 2017) provided identical Δ_47_ values (within the standard error of each measurement). Analytical details and carbonate reference materials are provided in the Supporting Information (SI1 and Tables S1–S5).

### Paleosols in the north alpine foreland basin

As the westernmost extension of the former Paratethys, the NAFB formed as a consequence of Alpine convergence. After deposition of Eocene to early Oligocene deep marine flysch sediments in the central part of the NAFB, Oligocene to middle Miocene (~32 to 11 Ma) sedimentation in the Swiss Molasse Basin is characterized by two large-scale coarsening-upward cycles with alternating shallow marine to freshwater/alluvial conditions^24,27,30^. The second coarsening-upward cycle, the transgression of the Upper Marine Molasse (OMM) and the transition to the terrestrial Upper Freshwater Molasse (OSM), resulted in the deposition of a roughly 100-m-thick succession of coarse alluvial conglomerates, fluvial sandstones and marls during the early to late Miocene. At ~17 Ma a fully terrestrial depositional system had developed with basin-axial, west-directed drainage systems. In the proximal part of the basin, close to the northern Alpine thrust front, large alluvial fan systems deposited south-derived sediments indicative of a northward drainage system (Fig. 1)^24,27,28^. The Fontannen/Napf (Switzerland) section sampled here is part of the Napf alluvial mega fan system, consisting of alternating conglomerates and sandstones as well as mudstones with abundant well-developed paleosols.

The Napf section has been dated by a combination of high-resolution magnetostratigraphy and biostratigraphy using micro-mammal faunas^29^. The original magnetostratigraphy has been partly revised^24,30^. Both revised versions assign the base of the section to Chron 5Dr (17.533–17.717 Ma; ages based on ref. ^31^) and the base of the youngest normal to chron 5ADn, but suggest different correlations of the youngest reversal: The youngest reversal can be correlated either to chron 5ABr (13.608–13.739 Ma) or to chron 5ACr (14.070–14.163 Ma)^24,30^. Assigning the youngest reversal of the section to chron 5ABr has been preferred by ref. ^30^, but the alternative age model is equally possible, because the uppermost micromammal fauna in the sections fauna places the top of the section only slightly above the Nördlinger Ries impact event (~14.81 Ma)^78^ and rather resembles MN5/MN6 (MN5: ~16.4–14.2 Ma, MN6: ~14.2–13.1/12.6 Ma) (not MN7/8: ~13.8(?)/13.1–11.2 Ma)^24^. Thus, we report both age models in Table 1, but only discuss the published age model, as it is the more conservative age constraint for describing the observed temperature shifts (minimum duration). Individual pedogenic carbonate nodules from mature paleosols were sampled along the same magnetostratigraphic section^29^, allowing the exact localization of each sample site within the magnetic pattern. The ages at the base and the top of each magnetozone are assigned to the middle distance between two juxtaposed sample sites of changed magnetic polarity. For samples that fall into one single magnetic pattern (i.e. between two assigned ages), the age constraints are based on a linear sedimentation rate. The uncertainty on the age determination corresponds to the sample spacing, which is on average 21.1 m (median 6.3 m) and translates into ~99 ka (~29 ka) using an average sedimentation rate of 0.21 mm/a, but varies with individual sample spacing and sedimentation rate within each chronozone.

## Supplementary information

Supporting Information.

Supplementary Dataset 1.

## Data Availability

All supporting datasets are available as Supplementary Information files that will be freely accessible on nature.com upon publication.
