# Supplementary material for: Middle Miocene long-term continental temperature change in and out of pace with marine climate records
Source: Sci Rep. 2020 May 14;10:7989. doi: 10.1038/s41598-020-64743-5 (PMC7224295; doi:10.1038/s41598-020-64743-5)
Supplement: Supplementary file 1 — Supporting Information. [file 41598_2020_64743_MOESM1_ESM.docx]

Supporting Information for

**Middle Miocene long-term continental temperature change in and out of pace with marine climate records**

Methner, Katharina^1,a^; Campani, Marion^1^; Fiebig, Jens^2^; Löffler, Niklas^1,2^; Kempf, Oliver^3^; Mulch, Andreas^1,2^

^1^Senckenberg Biodiversity and Climate Research Centre (BiK-F)

Senckenberganlage 25

60325 Frankfurt/Main, Germany

^2^Institut für Geowissenschaften

Goethe-Universität

Altenhöferallee 1

60438 Frankfurt/Main, Germany

^3^Bundesamt für Landestopografie swisstopo,

Geologische Landesaufnahme

Seftigenstr. 264

3084 Wabern, Switzerland

^a^Corresponding Author:

Katharina Methner

Senckenberg Biodiversity and Climate Research Centre (BiK-F)

Senckenberganlage 25

60325 Frankfurt/Main

0049 69 7542 1815

katharina.methner@senckenberg.de

**Contents of this file**

- - Text SI1
  - Figure SI1
  - Tables SI1 to S5

The supplementary information comprises text document S1, which describes the analytical procedure of stable isotope (δ^18^O, δ^13^C) and clumped isotope (Δ_47_) analyses and the calculation of oxygen isotopic compositions of water (δ^18^O_water_). It further contains an additional figure (stable isotope data, published in Campani et al. 2012) and 5 data tables (separate file). These tables contain the sample and age information, the analytical results of stable isotope (Tab. SI1) and of clumped isotope analyses (Tab. SI2 – SI5)..

SI1. Analytical procedure

1.1. Stable isotope analyses

High-resolution oxygen and carbon isotope analyses of carbonates were performed at Leibniz Universität Hannover, Germany and data were presented in Campani et al. (2012). Carbonate sample powders were drilled using a low speed dental drill. 0.2 mg to 1.0 mg of carbonate powder were digested with phosphoric acid at 72 °C in a sealed reaction vessel and flushed with helium gas. The evolved CO_2_ was sampled by a Thermo Scientific GasBench II and isotope ratios were measured on a Thermo Electron delta V advantage mass spectrometer. International and in-house standards were run in-line with the samples. NBS 19 yielded an average of δ^18^O = 28.60‰ (n = 34); the average uncertainty was ±0.2‰. All isotopic results are reported in standard delta notation and corrected to VSMOW (δ^18^O) or VPDB (δ^13^C) (Tab. SI1).

1.2. Clumped isotope analyses

Clumped isotope thermometry allows determination of carbonate formation temperatures. In equilibrium, the abundance of CO_2_ isotopologues containing bonds between heavy isotopes of oxygen and carbon is purely temperature-dependent. C-O bond abundances are thereby expressed as the Δ_47_ value, which mainly describes the difference of the abundance of the ^13^C^18^O^16^O-isotopologue of the sample gas (R^47^, R^46^, R^45^) from the theoretical stochastic distribution of the gas (R^47*^, R^46*^, R^45*^) with the same bulk isotopic composition (Eiler (2007, 2011) and ref. therein):

Δ_47_ = ((R^47^/ R^47*^ - 1) – (R^46^/ R^46*^ - 1) - (R^45^/ R^45*^ - 1)) * 1000 (‰) (ii)

with R^i^ = mass i / mass 44.

Carbonate powder (6**-**15 mg) was digested in >106% phosphoric acid at 90°C ± 0.1°C for 30 minutes. For carbonate digestion we used an automated acid bath as described in (Wacker et al., 2013) and (Fiebig et al., 2016). The CO_2_ cleaning procedure and measurements follow (Bajnai et al., 2018; Wacker et al., 2013, 2014). Briefly, the produced CO_2_ was purified by passing it through cryogenic traps (-80°C) before and after passage through a Porapak Q-packed gas chromatography column (-15°C) to remove traces of hydrocarbons. The cleaned CO_2_ was measured on a Thermo ScientificMAT 253 gas source isotope ratio mass spectrometer dedicated to the measurements of masses 44 to 49 at the joint Goethe University – Senckenbrg BiK-F Stable Isotope Facility at the Institute of Geosciences, Goethe University Frankfurt. Each measurement included ten acquisitions (consisting of ten cycles each with an ion integration time of 20 s).

CO_2_ equilibrated at 1000°C and 25°C were measured along with the samples to monitor the non-linearity of the mass spectrometer and the extent of scale compression (Tab. SI2). For samples analyzed prior to 2015, non-linearity was corrected using the heated gas approach of (Huntington et al., 2009). For samples analyzed in 2016/2017, measured m/z 47 intensities were directly corrected for the contribution of secondary electrons to m/z 47 after scaling the negative backgound on m/z 47 to the measured m/z 49 intensity (Fiebig et al., 2016). All data is reported in the CDES (Carbon Dioxide Equilibrium Scale) as proposed by Dennis et al. (2011). For this purpose, the theoretical 25°C-90°C acid fractionation factor of +0.069‰ (Guo et al., 2009) was applied. All data was processed using the Gonfiantini set of isotopic parameters (^13^R_PDB_ = 0.0112372, ^18^R_VSMOW_ = 0.0020052, ^17^R_VSMOW_ = 0.0003799 and λ = 0.5164).

Temperatures are calculated using the calibration of Wacker et al. (2014):

Δ_47_ = 0.0327 (±0.0026) * 10^6^/T^2^ + 0.3030 (±0.0308) (iii)

with Δ_47_ in ‰ and T in K. Within the analytical error, this regression line (iii) is statistically indistinguishable from (a) the empirically determined calibration line of (Henkes et al., 2013) (after applying a consistent 25°C-90°C acid fractionation factor to both data sets) and (b) the theoretical calibration line (Guo et al., 2009) (after applying a 25°C acid fractionation factor of 0.268‰ (Passey and Henkes, 2012) to equation (18) provided by Guo et al. (2009). We refer to calibration (iii) because it has been determined at Goethe University Frankfurt under similar analytical conditions (digestion at 90°C; similar gas preparation; all measurements performed at the same mass spectrometer).

Each day 2-3 carbonate reference materials were measured (Tab. SI4). These were Carrara marble and *Arctica islandica* (also referred to as MuStd, a well-homogenized shell material of an aragonitic cold water bivalve), ETH 1 and ETH 3. For *Arctica islandica* we receive a mean Δ_47_ value of 0.720‰ ± 0.006‰ (1 SE, n=24), indistinguishable from the 0.724‰ ±0.004‰ (1 SE, n=28) obtained by Wacker et al. (2014) during the time interval of their calibration. Carrara marble yields a mean Δ_47_ value of 0.376‰ ±0.002‰ (1 SE, n=58). This reference material was not analyzed by Wacker et al. (2014). Its mean value, however, is indistinguishable from those reported by other labs (e.g., Bonifacie et al., 2017; Dennis et al., 2011) after applying a unique 25°C-90°C acid fractionation factor of 0.069‰ to all data.

External standard errors (SE) for 4-5 replicate measurements range from ±0.002‰ to ±0.011‰. A straightforward way to determine an error based on the mass spectrometric setup exclusively (and not on sample characteristics) is the consideration of its shot noise limit (Merritt and Hayes, 1994). SE values < ~0.0040‰ (n=4) and < ~0.0036‰ (n=5) are below the shot noise limit of our mass spectrometric setup (10 acquisitions, consisting of 10 cycles at 20 s integration time each, represent one replicate measurement) after (Merritt and Hayes, 1994). Therefore, whenever 1 SE is smaller than 0.0040‰, we provide the respective shot noise limit of the mass spectrometer which represents the minimum error of a homogeneous sample measurement based on the analytical setup.

1.3. Soil water oxygen isotopic composition

We calculated the oxygen isotopic composition of water (δ^18^O_water_) from Δ_47_ temperatures and GasBench-obtained δ^18^O_carbonate_ values using oxygen isotope fractionation coefficients calculated after Kim and O'Neil (1997) that was updated new acid fractionation factors of Kim et al. (2007):

1000 ln α = 18.03 (10^3^*T^-1^) - 32.23 (iv)

where α is the oxygen isotope fractionation coefficient and T is the fractionation temperature in K. Note that the original intercept of -32.42 reported by Kim and O'Neil (1997) has been corrected by +0.19‰ which takes into account the difference in the 25°C acid fractionation factors of Kim et al. (2007) relative to Kim and O'Neil (1997). For clarity and to avoid temperature interpolations between sample localities, we only used calculated δ^18^O_water_ from those samples where we obtained clumped isotope analyses (Tab. SI5).

1.4. Extraction of climate model data from Zhou et al. (2018)

The approximate paleo-locality of the Fontannen section is at 7.5°E longitude and 44.5°N latitude at 15 Ma according to www.paleolatitute.org (lower bound 41.9° and upper bound 46.3°). We extracted climate model output from the closest grid cells of the paleogeographicla porsiotion of the Fontannen section (3x3 cells), namely, 5°, 7.5°, and 10°E and 46.4°, 44.5°, 42.6° N and for comparison 3x3 grid cells around the modern location of the section, namely, 5°, 7.5°, and 10°E and 48.3° 46.4°, 44.5°N. The extracted data include monthly 2m air temperature, ground temperatures, and the daily maximum of average 2m air temperatures as well as modelled relative humidity and soil water contents. We calculated the MAT, summer temperatures of JJA (likely closest to WMMT), winter temperatures of DJF (CMMT), the seasonal range of temperature (either warmest month vs. coldest month or JJA-DJF), and the differences between JJA temperature and MAT. This represents our suggested shift in temperature due to reorganization of rainfall patterns between the MCO and the MMCT. Results are described in the manuscript and are shown in Fig. SI3.

**Figures**


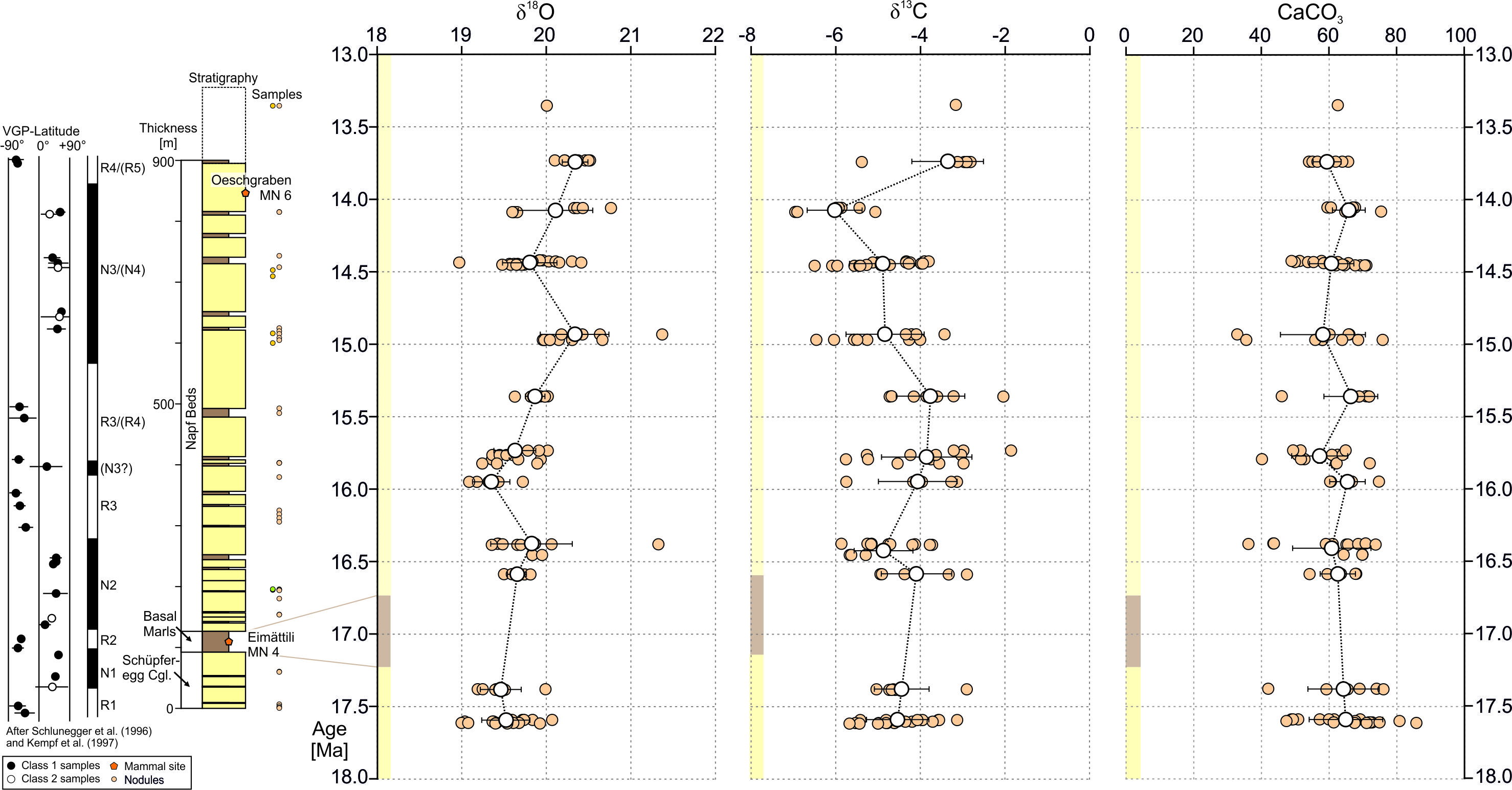


Figure SI1. Stratigraphic and paleomagnetic section (Schlunegger et al. (1996)). Age assignments are based on Kempf et al. (1997). All stable isotope data has been acquired by M. Campani (SBiK-F), δ^18^O data is published in Campani et al. (2012).


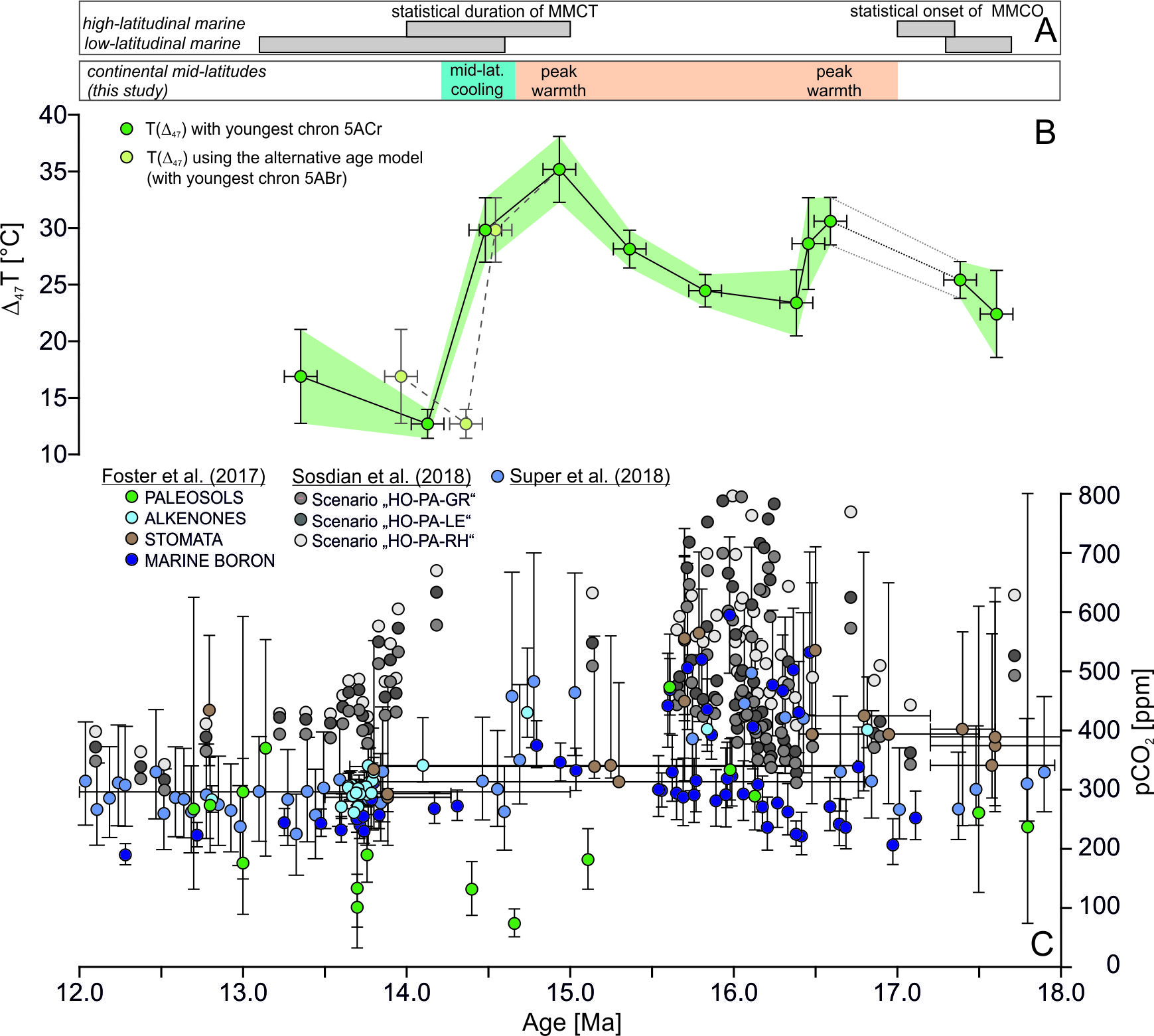


**Figure SI2**. (A) Age constraints of the statistical onset of the MCO start and the duration of the MMCT (Mudelsee et al. (2014)). (B+C) Comparison of terrestrial soil carbonate temperatures (T(Δ_47_)) (B) with pCO_2_ reconstructions (C), using the compilation of Foster et al. (2017) and new estimates of Super et al. (2018) and Sosdian et al. (2018). The alternative age model is indicated by the light green data in (B); ages are given in the manuscript Tab. 1.

**
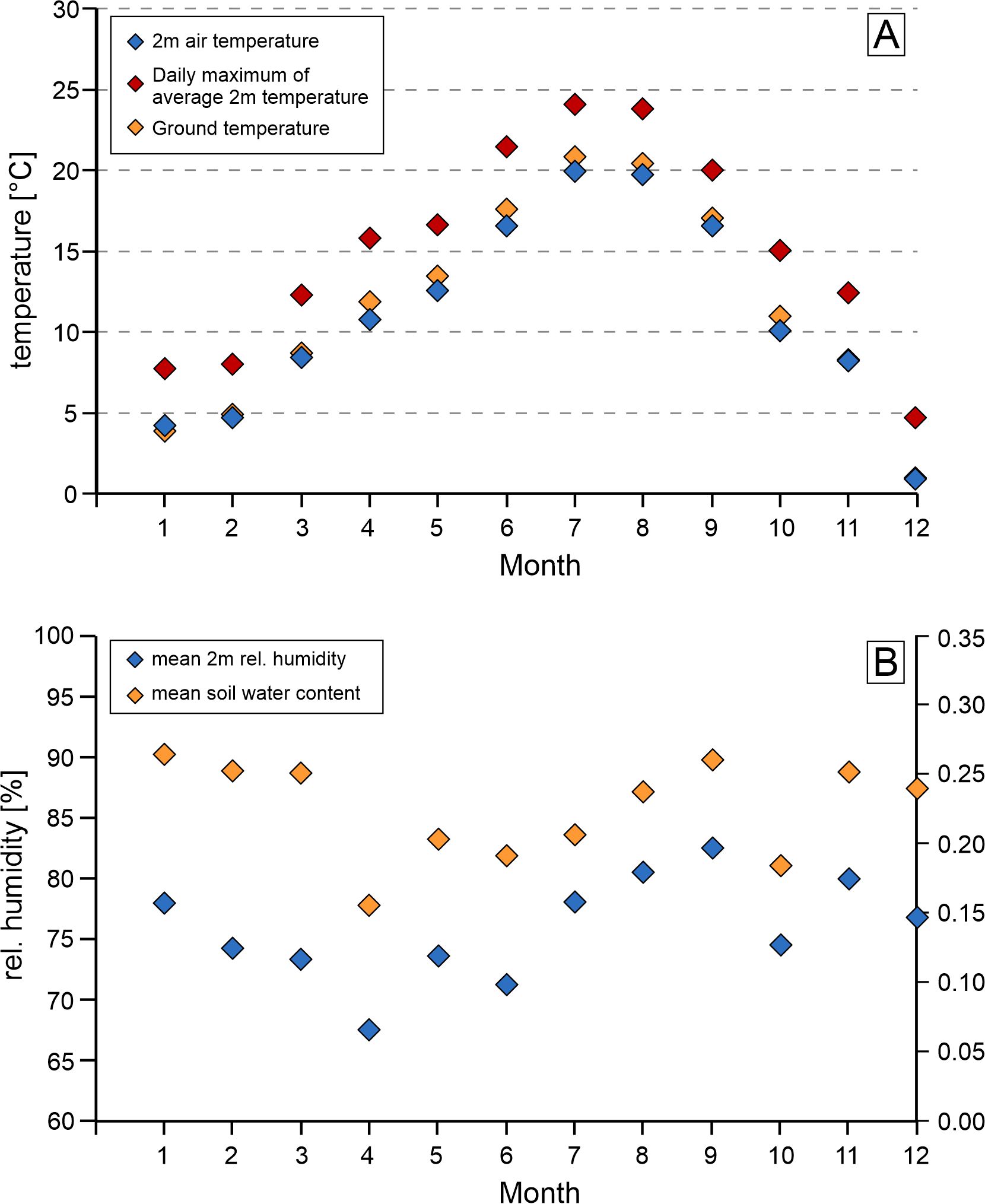
**

**Fig. SI3.** (A) Modeled temperature data including monthly 2m air temperature (blue diamonds), ground temperatures (green diamonds) and the daily maximum of average 2m air temperatures (red diamonds). (B) Modelled relative humidity (blue diamonds) and soil water (green diamonds) contents. Data from Zhou et al. (2018).

**Tables (in separate file)**

**Table SI1.** Pedogenic carbonate sample information (coordinates in Swiss coord. system, stratigraphic position, assigned ages) with GasBench oxygen (δ^18^O, VSMOW, ‰) and carbon (δ^13^C, VPDB, ‰) isotope ratios and calculated CaCO_3_ content (%wt.). Different subsamples (e.g. MC954A to C) are nodules from the same bed. All data has been acquired by M. Campani, δ^18^O data is published in Campani et al. (2012).

**Table SI2.** Measured δ^47^ and Δ_47_ values (in ‰) of “heated gases” (equilibrated at 1000°C) and CO_2_ gases equilibrated at 25 °C for the time intervals of sample measurements.

**Table SI3.** Empirical transfer function (ETF) applied during this study with x: background corrected Δ_47_ value and y: Δ_47 CDES_ (CDES: Carbon dioxide equilibrium scale; Dennis et al., 2011)).

**Table SI4.** Measured isotopic compositions (δ^47^, Δ_47_, δ^13^C and δ^18^O values in ‰) of standard materials. Each day, 2-3 in-house standard materials were measured: Carrara marble, *Arctica islandica* ("MuStd", well-homogenized shell material of an aragonitic cold water bivalve), as well as ETH-1 and ETH-3.

**Table SI5.** Measured isotopic compositions (δ^47^, Δ_47_, δ^13^C and δ^18^O values in ‰) of pedogenic carbonate samples and calculated temperatures using the calibration of Wacker et al. (2014). Since 2014, δ^47^ raw, Δ_47_ raw values are corrected using the background correction of Fiebig et al. (2016).

**References**

Bajnai, D., Fiebig, J., Tomašových, A., Milner Garcia, S., Rollion-Bard, C., Raddatz, J., Löffler, N., Primo-Ramos, C. and Brand, U. (2018) Assessing kinetic fractionation in brachiopod calcite using clumped isotopes. Scientific Reports 8, 533.

Bonifacie, M., Calmels, D., Eiler, J.M., Horita, J., Chaduteau, C., Vasconcelos, C., Agrinier, P., Katz, A., Passey, B.H., Ferry, J.M. and Bourrand, J.-J. (2017) Calibration of the dolomite clumped isotope thermometer from 25 to 350 °C, and implications for a universal calibration for all (Ca, Mg, Fe)CO3 carbonates. Geochim Cosmochim Ac 200, 255-279.

Campani, M., Mulch, A., Kempf, O., Schlunegger, F. and Mancktelow, N. (2012) Miocene paleotopography of the Central Alps. Earth Planet Sc Lett 337–338, 174-185.

Dennis, K.J., Affek, H.P., Passey, B.H., Schrag, D.P. and Eiler, J.M. (2011) Defining an absolute reference frame for ‘clumped’ isotope studies of CO_2_. Geochim Cosmochim Ac 75, 7117-7131.

Eiler, J.M. (2007) “Clumped-isotope” geochemistry—The study of naturally-occurring, multiply-substituted isotopologues. Earth Planet Sc Lett 262, 309-327.

Eiler, J.M. (2011) Paleoclimate reconstruction using carbonate clumped isotope thermometry. Quaternary Sci Rev 30, 3575-3588.

Fiebig, J., Hofmann, S., Löffler, N., Lüdecke, T., Methner, K. and Wacker, U. (2016) Slight pressure imbalances can affect accuracy and precision of dual inlet-based clumped isotope analysis. Isot Environ Healt S 52, 12-28.

Foster, G.L., Royer, D.L., Lunt, D.J., 2017. Future climate forcing potentially without precedent in the last 420 million years. Nature Communications 8, 14845, doi:10.1038/ncomms14845.

Guo, W., Mosenfelder, J.L., Goddard Iii, W.A. and Eiler, J.M. (2009) Isotopic fractionations associated with phosphoric acid digestion of carbonate minerals: Insights from first-principles theoretical modeling and clumped isotope measurements. Geochim Cosmochim Ac 73, 7203-7225.

Henkes, G.A., Passey, B.H., Wanamaker Jr, A.D., Grossman, E.L., Ambrose Jr, W.G. and Carroll, M.L. (2013) Carbonate clumped isotope compositions of modern marine mollusk and brachiopod shells. Geochim Cosmochim Ac 106, 307-325.

Huntington, K.W., Eiler, J.M., Affek, H.P., Guo, W., Bonifacie, M., Yeung, L.Y., Thiagarajan, N., Passey, B., Tripati, A., Daëron, M. and Came, R. (2009) Methods and limitations of ‘clumped’ CO_2_ isotope (Δ_47_) analysis by gas-source isotope ratio mass spectrometry. Journal of Mass Spectrometry 44, 1318-1329.

Kempf, O., Bolliger, T., Kälin, D., Engesser, B. and Matter, A. (1997) New magnetostratigraphic calibration of Early to Middle Miocene mammal biozones of the North Alpine foreland basin. Mémoires et travaux de l'Institut de Montpellier, 547-561.

Kim, S.-T., Mucci, A. and Taylor, B.E. (2007) Phosphoric acid fractionation factors for calcite and aragonite between 25 and 75 C: revisited. Chem Geol 246, 135-146.

Kim, S.-T. and O'Neil, J.R. (1997) Equilibrium and nonequilibrium oxygen isotope effects in synthetic carbonates. Geochim Cosmochim Ac 61, 3461-3475.

Merritt, D.A. and Hayes, J.M. (1994) Factors Controlling Precision and Accuracy in Isotope-Ratio-Monitoring Mass Spectrometry. Anal Chem 66, 2336-2347.

Mudelsee, M., Bickert, T., Lear, C.H., Lohmann, G., 2014. Cenozoic climate changes: A review based on time series analysis of marine benthic δ18O records. Rev Geophys 52(3), 333-374, doi:10.1002/2013RG000440.

Passey, B.H., Henkes, G.A., 2012. Carbonate clumped isotope bond reordering and geospeedometry. Earth Planet Sc Lett 351–352, 223-236, doi:10.1016/j.epsl.2012.07.021.

Schlunegger, F., Burbank, D., Matter, A., Engesser, B., Modden, C., 1996. Magnetostratigraphic calibration of the Oligocence to Middle Miocene (30-15 Ma) mammal biozones and depositional sequences of the Swiss Molasse basin. Eclogae Geologicae Helvetiae 89(2), 753-788, doi:10.5169/seals-167923.

Sosdian, S.M., Greenop, R., Hain, M.P., Foster, G.L., Pearson, P.N., Lear, C.H., 2018. Constraining the evolution of Neogene ocean carbonate chemistry using the boron isotope pH proxy. Earth Planet Sc Lett 498, 362-376, doi:10.1016/j.epsl.2018.06.017.

Super, J.R., Thomas, E., Pagani, M., Huber, M., O’Brien, C., Pincelli, M.H., 2018. North Atlantic temperature and pCO2 coupling in the early-middle Miocene. Geology 46(6), 519-522, doi:10.1130/G40228.1.

Wacker, U., Fiebig, J. and Schoene, B.R. (2013) Clumped isotope analysis of carbonates: comparison of two different acid digestion techniques. Rapid Communications in Mass Spectrometry 27, 1631-1642.

Wacker, U., Fiebig, J., Tödter, J., Schöne, B.R., Bahr, A., Friedrich, O., Tütken, T., Gischler, E. and Joachimski, M.M. (2014) Empirical calibration of the clumped isotope paleothermometer using calcites of various origins. Geochim Cosmochim Ac 141, 127-144.

Zhou, H., Helliker, B.R., Huber, M., Dicks, A., Akçay, E. (2018) C4 photosynthesis and climate through the lens of optimality. Proceedings of the National Academy of Sciences 115, 12057-12062, doi:10.1073/pnas.1718988115.
